# Role of AmiA in the Morphological Transition of Helicobacter pylori and in Immune Escape

**DOI:** 10.1371/journal.ppat.0020097

**Published:** 2006-09-22

**Authors:** Catherine Chaput, Chantal Ecobichon, Nadège Cayet, Stephen E Girardin, Catherine Werts, Stéphanie Guadagnini, Marie-Christine Prévost, Dominique Mengin-Lecreulx, Agnès Labigne, Ivo G Boneca

**Affiliations:** 1 Unité de Pathogénie Bactérienne des Muqueuses, Institut Pasteur, Paris, France; 2 Plate-Forme de Microscopie Électronique, Institut Pasteur, Paris, France; 3 Groupe Avenir, INSERM U389, Unité de Pathogénie Microbienne Moléculaire, Institut Pasteur, Paris, France; 4 Groupe Immunité Innée et Signalisation, Institut Pasteur, Paris, France; 5 Enveloppes Bactériennes et Antibiotiques, UMR 8619 CNRS, Université Paris-Sud, Orsay, France; Washington University School of Medicine, United States of America

## Abstract

The human gastric pathogen Helicobacter pylori is responsible for peptic ulcers and neoplasia. Both in vitro and in the human stomach it can be found in two forms, the bacillary and coccoid forms. The molecular mechanisms of the morphological transition between these two forms and the role of coccoids remain largely unknown. The peptidoglycan (PG) layer is a major determinant of bacterial cell shape, and therefore we studied H. pylori PG structure during the morphological transition. The transition correlated with an accumulation of the *N*-acetyl-D-glucosaminyl-β(1,4)-*N*-acetylmuramyl-L-Ala–D-Glu (GM-dipeptide) motif. We investigated the molecular mechanisms responsible for the GM-dipeptide motif accumulation, and studied the role of various putative PG hydrolases in this process. Interestingly, a mutant strain with a mutation in the *amiA* gene, encoding a putative PG hydrolase, was impaired in accumulating the GM-dipeptide motif and transforming into coccoids. We investigated the role of the morphological transition and the PG modification in the biology of H. pylori. PG modification and transformation of H. pylori was accompanied by an escape from detection by human Nod1 and the absence of NF-κB activation in epithelial cells. Accordingly, coccoids were unable to induce IL-8 secretion by AGS gastric epithelial cells. *amiA* is, to our knowledge, the first genetic determinant discovered to be required for this morphological transition into the coccoid forms, and therefore contributes to modulation of the host response and participates in the chronicity of H. pylori infection.

## Synopsis

Helicobacter pylori is a human pathogen responsible for gastric diseases such as ulcers and gastric cancers. Despite the host's vigorous immune response, H. pylori is capable of persisting for decades in its human host. H. pylori is found in biopsies in two distinct forms, a spiral rod form and a coccoid form. Chaput et al. investigated the molecular mechanisms leading to the transition of H. pylori from a spiral rod–shaped organism to a coccoid organism. The morphological transition is accompanied by modifications of the bacterial cell wall peptidoglycan. The authors have identified the AmiA protein as essential for this morphological transition and modification of the cell wall peptidoglycan. Additionally, the authors show that the cell wall modifications and morphological transition allow these coccoid forms to escape detection by the immune system and therefore could participate in the persistence of H. pylori infection during the lifetime of its human host.

## Introduction

Helicobacter pylori is a human pathogen with an unique niche: the stomach. The presence of this bacterium is always associated with chronic gastritis, and less often with severe duodenal ulcers, gastric adenocarcinoma, or mucosa-associated lymphoid tissue lymphoma. H. pylori has the interesting ability to convert from bacillary to coccoid forms. The coccoid forms appear in stationary phase and can also be induced under stress conditions, for example, following modification of pH, O_2_ tension, or temperature [1,2], or exposure to antibiotics such as amoxicillin [3,4]. However, there is still controversy regarding the biological role of this form. Both forms are commonly observed in the human stomach [5,6]. Coccoids are viable but noncultivable, and this has led to the suggestion that the coccoid form is the persisting form, allowing H. pylori to spread between human hosts. Coccoid forms contain a reasonable quantity of ATP [7] and an active respiratory chain [8–10]; it is also viable as assessed by viability staining [11–14]. Various proteins (including VacA and CagA) and activities (for example, urease activity) are detectable, but it is not clear whether there is any de novo protein synthesis [15]. Attempts to revert coccoid bacteria to spiral under laboratory conditions have failed so far. In contrast, several groups have reported colonization of mice with coccoid bacteria and have subsequently isolated spiral bacteria from their stomachs, indicating that under certain conditions coccoids may revert back to spiral bacteria [16–19].

Despite interest in this subject, little is known about the process of morphological transition into coccoid forms. Proteome and transcriptome analyses have failed to identify proteins involved in the transition [7,20–23]. The *cdrA* gene has been implicated in coccoid formation [24], but these results are controversial because the *cdrA* gene is inactivated in several strains, including the two sequenced strains 26695 and J99. Hence, CdrA is unlikely to have a major role, if any, in coccoid formation. It is known, however, that the lipid composition of H. pylori changes substantially during the transition into coccoid forms [25].

One of the main determinants of bacterial shape is the peptidoglycan (PG) layer (for a recent review see [26]). Costa et al. [27] implicated a modification of the muropeptide composition of H. pylori PG in the transition from the bacillary to the coccoid form: the *N*-acetyl-D-glucosaminyl-β(1,4)-*N*-acetylmuramyl-L-Ala–D-Glu (GM-dipeptide) motif accumulated in the sacculus after 2 d of liquid culture. This motif lacks the diamino acid, *meso*-diaminopimelic acid (*meso*DAP), required for PG transpeptidation. Possibly, a change to a looser PG macromolecule could explain the shape transition of H. pylori from spiral to coccoid.

Here, we studied the genetic determinants involved in the accumulation of the GM-dipeptide motif. Several alternative mechanisms could explain this phenomenon (see Protocol S1 and Figure S2), and PG hydrolases could be involved. We describe the construction of a mutant strain with a mutation of the *amiA* gene—encoding a putative PG hydrolase—that is impaired in the accumulation of the GM-dipeptide motif; it is also defective in the transition from spiral bacteria into coccoid forms. We show that the phenotype of morphological transition and PG modification is associated with impaired sensing by the Nod1 pathway, impaired activation of NF-κB, and impaired cytokine production by AGS gastric epithelial cells. We thus identified a new mechanism for bacterial escape from the innate immune system.

## Results

### Accumulation of the GM-Dipeptide Motif in the PG of Various Strains of H. pylori

**Figure 1:**
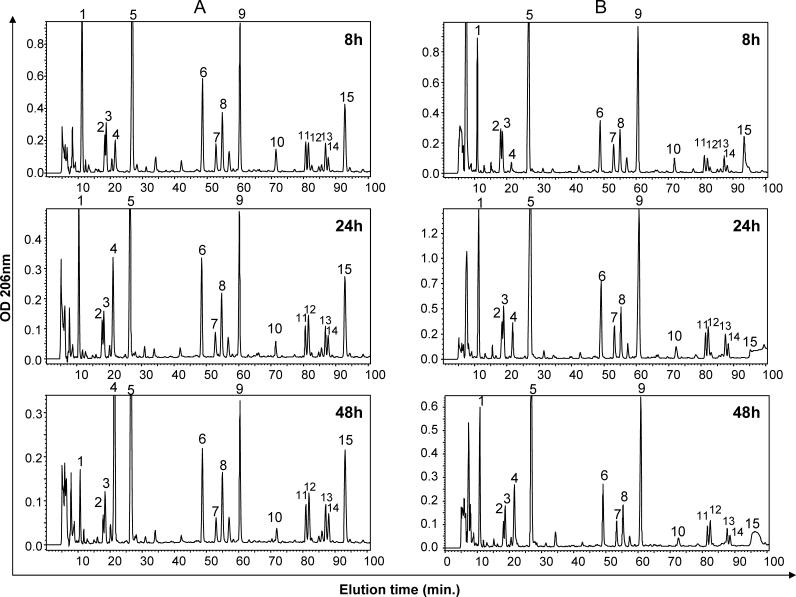
Muropeptide Profile of H. pylori PG PG from parental strain 26695 (A) and its *amiA* isogenic mutant (B) were purified and digested with the muramidase M1 (mutanolysin). The generated muropeptides were separated by HPLC. The HPLC profiles show muropeptide composition after 8 h, 24 h, and 48 h of bacterial growth. Each peak structure was assigned by MALDI-TOF mass spectrometry and corresponds to a different muropeptide: (1) GM-tripeptide, (2) GM-tetrapeptide, (3) GM-tetrapeptide-glycine, (4) GM-dipeptide, and (5) GM-pentapeptide. Dimers were then eluted: (6) GM-tetrapeptide-tripeptide-MG, (7) GM-tetrapeptide-tetrapeptide-glycine-MG, (8) GM-tetrapeptide-tetrapeptide-MG, and (9) GM-tetrapeptide-pentapeptide-MG. Finally, anhydromuropeptides were eluted: (10) G(anh)M-pentapeptide, (11) and (12) G(anh)M-tetrapeptide-tripeptide-MG, (13) and (14) G(anh)M-tetrapeptide-tetrapeptide-MG, and (15) G(anh)M-tetrapeptide-pentapeptide-MG.

We purified and analyzed the PG from the sequenced strain 26695 and from the strain NCTC11637 used as a control. No major difference between chromatograms of the two strains was observed (Figures 1 and S1). Muropeptide composition analysis of H. pylori PG showed an accumulation of the GM-dipeptide motif in strain 26695 during the stationary phase, as previously observed in strain NCTC11637 (Figures 1 and S1; [27]). Interestingly, the accumulation of the GM-dipeptide (peak 4 in Figures 1 and S1) coincided with a decrease of *N*-acetyl-D-glucosaminyl-β(1,4)-*N*-acetylmuramyl-L-Ala–γ-D-Glu-*meso*DAP (GM-tripeptide) (peak 1).

We used a targeted approach to investigate the molecular mechanisms responsible for the accumulation of the GM-dipeptide motif (see Protocol S1 and Figure S2). We constructed mutants of *hp0087* (encoding a putative peptidase), *hp1118* (encoding a gamma-glutamyltranspeptidase), *hp0645* (encoding the lytic transglycosylase Slt), *hp1572* (encoding the lytic transglycosylase MltD), and *hp0772* (encoding the putative *N*-acetylmuramoyl-L-alanine amidase AmiA). Detailed information for each gene and protein is available on the PyloriGene database (http://genolist.pasteur.fr/PyloriGene/genome.cgi). Only in the *amiA* mutant was the accumulation of GM-dipeptide impaired (peak 4 in Figures 1 and S3). The PG of this mutant contained less of this motif at 8 h, 24 h, and 48 h (about 1.9-, 2.3-, and 2.9-fold less, respectively) than the parental strain (Figure 1). The amount of GM-tripeptide (peak 1) remained stable between exponential and stationary phase. The residual amount of GM-dipeptide present in the PG of the *amiA* mutant is probably due to the decrease of MurE activity in stationary phase (Figure S4).

### Morphology of the *amiA* Mutant

**Figure 2:**
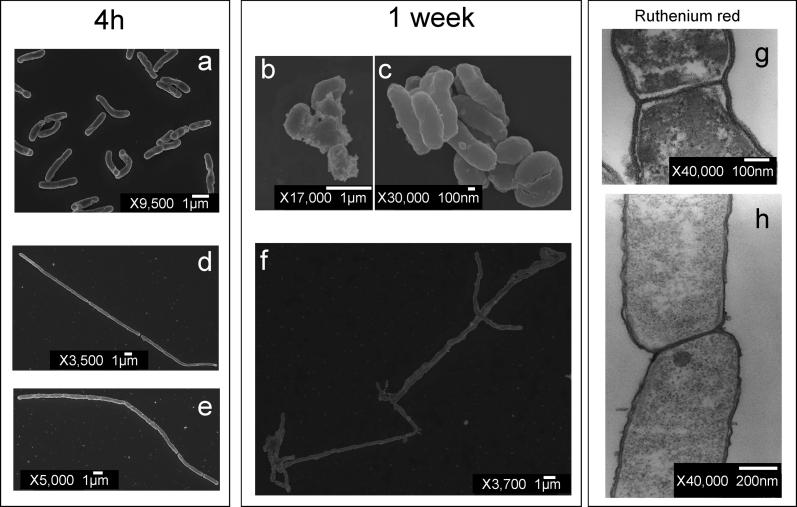
Morphologies of H. pylori (A–F) SEM of H. pylori during exponential-phase growth (4 h of culture (A, D, and E) and after 1 wk of culture (B, C, and F) of the parental strain 26695 (A–C) and the *amiA* mutant (D–F). (G and H) TEM sections of the *amiA* mutant after ruthenium staining. The *amiA* mutant is able to form a complete septum without final daughter cell separation. Chains of the *amiA* mutant contained up to 30–40 bacteria.

We studied the morphology of the *amiA* mutant during the different growth stages using scanning electron microscopy (SEM) and after ruthenium red staining using transmission electron microscopy (TEM) to visualize PG in the periplasmic space) (Figure 2). The *amiA* mutant was observed as very long bacterial chains of up to 30 bacteria per chain after 4 h of culture (Figure 2D and 2E), while the parental strain 26695 showed normal individual rod-shaped bacteria (Figure 2A). Sections stained with ruthenium red revealed completely formed septa in the *amiA* mutant (Figure 2G and 2H), indicating daughter cell separation was defective. The parental strain, 26695, showed rod, U, donut, and coccoid forms after 2 d, 1 wk, and 1 mo of culture (Figure 2B and 2C; unpublished data), while the *amiA* mutant remained in long chains of rod-shaped bacteria (Figure 2F). Far fewer *amiA* mutant cells were in coccoid forms after similar times of growth (Table 1). Therefore, the *amiA* mutant seems to be blocked both for cell separation and for the transition into coccoid forms.

**Table 1:**
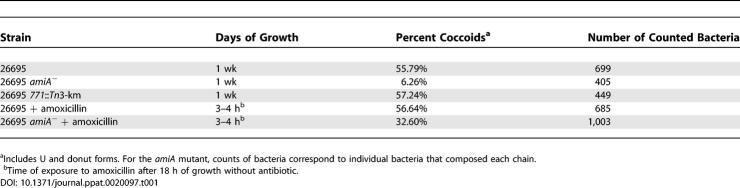
Quantification of the Number of Coccoid Forms

### Complementation of the *amiA* Mutant

Next, we tried to complement the phenotype by introducing a wild-type *amiA* gene at a different locus, that of the *rdxA* gene (Figure S5). Disruption of the *rdxA* gene confers metronidazole (mtz) resistance to H. pylori [28]. However, the insertion of a copy of the *amiA* gene into the *rdxA* gene in the same orientation was lethal for H. pylori. When the *amiA* gene was inserted into the *rdxA* gene in the opposite orientation, transformants were obtained. PCR analysis showed two populations of transformants: (1) bacteria with *amiA* in *rdxA* and the wild-type *amiA* gene inactivated by the kanamycin (km) cassette (mtz^R^km^R^ mutants), and (2) mutants with *amiA* in *rdxA* and with the wild-type *amiA* gene restored (mtz^R^km^S^ mutants). Only the second type of mutants (mtz^R^km^S^) complemented the filamentation phenotype and restored the transition into coccoid forms. Hence, the observed phenotype could not be due to a secondary mutation. To eliminate the possibility of polar effects of the *amiA* mutant on the downstream gene, we also constructed a mutant of the downstream gene, *hp0771*. The *hp0771* mutant showed a normal bacillary form during the first day of culture, and the capacity to adopt the coccoid form. We quantified the proportions of bacillary and coccoid forms (Table 1): the *amiA* mutant was the only strain impaired in the transition into coccoid forms.

### Amoxicillin Effects

**Figure 3:**
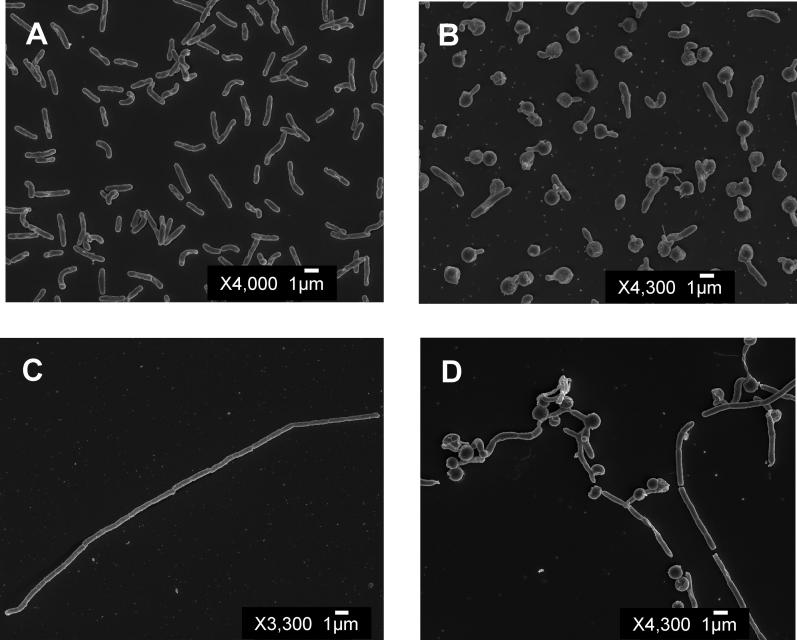
Effect of Amoxicillin on H. pylori Morphology SEM of H. pylori strain 26695 (A and B) and its isogenic *amiA* mutant (C and D) grown without amoxicillin (A and C) and after 3–4 h exposure to 10 μg/ml amoxicillin (B and D). Amoxicillin treatment of the *amiA* mutant bypasses the requirement of *amiA* for the morphological transition, indicating that absence of coccoid forms was not due to sterical hindrance of the bacterial chains.

Some stress signals, including amoxicillin treatment, can induce the morphological transition into coccoid forms [4]. We investigated the response of the *amiA* mutant to amoxicillin. First, we determined the minimum inhibitory concentration (MIC) of amoxicillin: it was identical for the *amiA* mutant and the parental strain 26695 (0.06 μg/ml). After overnight culture, 10 μg/ml amoxicillin was added to the medium, and after 3 h of antibiotic treatment, bacteria were observed using SEM. The *amiA* mutant formed chains of spherical bacteria (Figure 3), rod-shaped bacteria, and, most frequently, both rod-shaped and spherical bacteria. Thus, the impaired morphological transition is not an artifact and does not result from steric hindrance of bacterial chain formation (see Table 1 for quantification). Therefore, AmiA is required both for PG modifications and for the morphological transition.

### Epithelial Cell Response to H. pylori PG and Coccoid Forms

Having demonstrated that the transition into coccoid forms is a process controlled by AmiA, we investigated the biological role of the coccoid forms. The accumulation of the GM-dipeptide motif (Figure 1, peak 4) correlated with the almost disappearance of the GM-tripeptide motif (Figure 1, peak 1). These two muropeptides are the agonists of the human Nod2 (hNod2) and human Nod1 (hNod1) proteins, respectively [29]. Sensing of H. pylori PG by Nod1 is essential for the inflammatory response by gastric epithelial cells [30]. Therefore, the switch from being a hNod1 agonist to being a hNod2 agonist during coccoid formation could affect the ability of gastric epithelial cells to detect H. pylori and to develop an inflammatory response.

**Figure 4:**
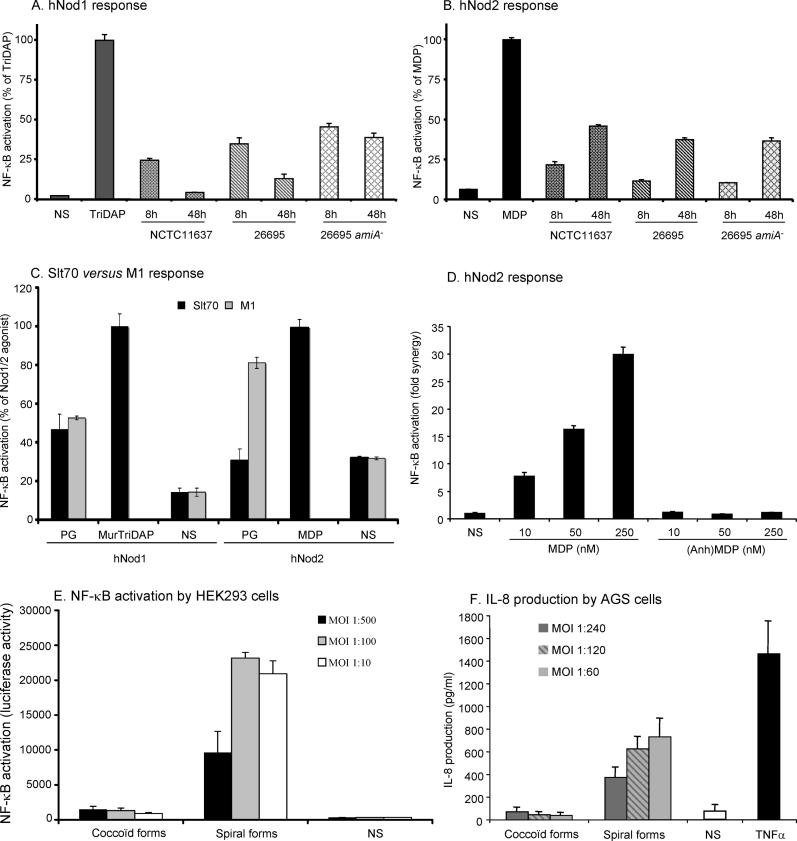
hNod1- and hNod2-Dependent Activation of NF-κB by H. pylori PG (A and B) PG samples from strain NCT11637, strain 26695, and the isogenic *amiA* mutant prepared after 8 h and 48 h of growth, were digested with M1 (mutanolysin) to generate muropeptides and used to stimulate hNod1 (A) and hNod2 (B). (C and D) PG samples were also digested with recombinant Slt70 from E. coli to generate anhydromuropeptides, used to stimulate hNod1 and hNod2, and compared to M1-generated muropeptides (C). hNod1 and hNod2 agonists were used at 10 nM and PGs at 0.3 μg/ml. Finally, purified GM-dipeptide and its anhydrous derivative G(anh)M-dipeptide were also tested for their ability to stimulate hNod2 (D). (E) H. pylori at different growth stages (spiral versus coccoid) and different multiplicity of infection (MOI) were used to stimulate the HEK293T cells, and NF-κB activation was determined. (F) The same experiment as in (E) was performed with the AGS gastric epithelial cell line, and IL-8 secretion was determined. TNF-α (20 ng/ml) was used as a positive control. MDP, muramyldipeptide; NS, nonstimulated; TriDAP, L-alanyl-D-glutamyl-*meso*DAP.

NF-κB activation in HEK293T cells via stimulation by hNod1 and hNod2 was tested with digested PG extracted from the *amiA* mutant and the parental strain after 8 h and 48 h of growth (Figure 4A). Nod1 responses showed highest NF-κB activation with PG extracted after 8 h of growth and less activation with PG extracted at 48 h of growth, for both wild-type strains (26695 and NCTC11637). Thus, the activation decreased with decreasing abundance of the GM-tripeptide in H. pylori PG. For the *amiA* mutant, hNod1 responses were the same when cells were stimulated with PG extracted after 8 h or 48 h of growth, consistent with the unchanging GM-tripeptide content of the PG. Conversely, hNod2 responses revealed a higher NF-κB activation with PG extracted after 48 h of growth than with PG extracted after 8 h (Figure 4B). These results suggest that spiral bacteria preferentially induce NF-κB via hNod1 and coccoid bacteria via hNod2.

However, hNod2 (as hNod1) senses muropeptides and not polymeric PG; we therefore tested whether naturally occurring PG turnover products can stimulate hNod2. These products are mainly anhydromuropeptides generated by endogenous PG hydrolases called lytic transglycosylases. We compared the hNod2-dependent activation of NF-κB by H. pylori PG digested by a muramidase (M1) and a lytic transglycosylase (Slt70 from Escherichia coli). Figure S6 shows the chromatogram of the Slt70-digested PG of H. pylori and the structural assignment of each anhydromuropeptide. As expected from our previous results [31], anhydromuropeptides were able to induce NF-κB in a Nod1-dependent manner (Figure 4C). Surprisingly, anhydromuropeptides were unable to induce NF-κB in a Nod2-dependent manner. To further investigate the structural basis of hNod2 sensing, we compared the Nod2-dependent activation of NF-κB by the GM-dipeptide and its anhydro derivative, G(anh)M-dipeptide ([anh]M indicates *N*-acetyl-anhydromuramic acid). The GM-dipeptide motif produced by H. pylori was detected via hNod2 in a dose-dependent manner. However, Nod2 did not sense the GanhM-dipeptide motif (Figure 4D). We conclude that PG turnover products are agonists of the Nod1 pathway [31], but are unable to induce the Nod2 pathway. Accordingly, rod-shaped H. pylori induced NF-κB in HEK293T cells and IL-8 production by gastric epithelial cells, but coccoid bacteria had no NF-κB or IL-8 stimulatory activities (Figure 4E and 4F). As epithelial cells do not respond to coccoid forms or to PG turnover products from coccoid forms, our study suggests that coccoid forms provide a route for immune escape for H. pylori.

## Discussion

Since the first observation of microbes, bacterial shape has been considered to be largely invariant and a characteristic feature of each species. It has therefore been used as a major taxonomic determinant. Nevertheless, several bacteria are known to change morphology during genetic developmental programs such as sporulation or asymmetric cell division. H. pylori undergoes morphological transition from spiral to coccoid. Previous attempts to identify specific markers or a dedicated genetic program involved in this morphological transition have been inconclusive [7,20–23]. Nevertheless, in 1999, Costa and colleagues correlated the morphological transition with a modification of H. pylori PG muropeptide composition [27], that is, the accumulation of the GM-dipeptide motif.

The PG layer is a major determinant of bacterial cell shape, so we felt that identifying the genetic determinants involved in the observed PG modification could help elucidate the morphological transition. There are several possible explanations for the accumulation of the GM-dipeptide motif (see Protocol S1 and Figure S2). It could result from a defect in precursor synthesis in the cytoplasm due to (1) a decrease of MurE activity blocking PG precursor synthesis at the step where *meso*DAP is added to the uracyl diphosphate-M-dipeptide, (2) insufficient *meso*DAP to allow synthesis of precursors, or (3) the presence of a carboxy/endopeptidase, cleaving between the second and the third amino acid residue. This carboxy/endopeptidase activity could be in either the cytoplasm (cleaving PG precursors with more than two amino acid residues in the stem peptide) or the periplasm (directly cleaving macromolecular PG). We also considered the potential roles of the annotated PG hydrolases Slt, MltD, and AmiA in this process [31]. Protocol S1 summarizes the various hypotheses and the data supporting or inconsistent with each of them. We identified the *amiA* gene as necessary for the PG modification. The *amiA* mutant was impaired in the transition to coccoid forms. This is, to our knowledge, the first identification of a genetic determinant required for the morphological transition of *H. pylori,* and also directly implicates PG modification in determining bacterial morphology. To our knowledge, this is the first description of a putative PG hydrolase directly involved in maintenance of bacterial cell shape. *N*-acetylmuramoyl-L-alanine amidases contribute to the separation of daughter cells in E. coli [32], but three genes encoding amidases had to be deleted from *E. coli* to observe a changed phenotype, whereas in H. pylori inactivation of a single gene was sufficient to produce a comparable filamentation phenotype.

Interestingly, the accumulation of GM-dipeptide motif (Figure 1, peak 4) coincided with a proportional decrease of the GM-tripeptide motif (Figure 1, peak 1). In the *amiA* mutant, the proportion of GM-tripeptide remained stable and the amounts of the GM-dipeptide were very low. No significant changes were observed for the other monomeric muropeptides. This is consistent with the activity of a periplasmic carboxy/endopeptidase that recognizes the γ-D-glutamyl-*meso*-diaminopimelic acid bond.

The AmiA protein is structured as a bimodular protein: a signal peptide followed by an N-terminal domain without homology to any sequences in the NCBI non-redundant database (amino acids 1–177), a linker peptide of variable length composed of KKEIP repeats (amino acids 178–190), and an C-terminal domain (amino acids 191–440) homologous to CwlU and CwlV, which are predicted to have an *N*-acetylmuramoyl-L-alanine amidase activity [33]. PG amidases cleave the PG in the periplasm between the *N*-acetylmuramic acid residue and the first amino acid residue of the peptide moiety, L-alanine. However, the amidase activity of AmiA and its closest homologs has never been confirmed, so it is plausible that AmiA has a carboxy/endopeptidase activity. Alternatively, AmiA might be bifunctional, with an N-terminal carboxy/endopeptidase activity and a C-terminal amidase activity. It is also possible that AmiA has an amidase activity that is unable to cleave stem peptides with less than three amino acid residues such as the human serum amidase or peptidoglycan recognition protein L [34]. This would lead to the elimination of stem peptides with three to five amino acid residues, and consequently the accumulation of GM-dipeptides. We are currently studying the biochemistry of the AmiA protein to resolve this issue.

We have shown that the morphological transition is regulated by AmiA. In its absence, the transition can be induced by treatment with amoxicillin, a β-lactam antibiotic. Exposure to amoxicillin bypasses the requirement for the AmiA protein, suggesting that one of the other determinants might be a penicillin-binding protein. Amoxicillin preferentially targets H. pylori PBP2 [4], a homolog of E. coli PBP2. A PBP2 conditional mutant of E. coli becomes spherical at nonpermissive temperature, [35] and, consequently, PBP2 is believed to drive lateral PG synthesis.

The role of PG metabolism in the transition into coccoid forms suggests this might be a regulated process rather than a random degeneration of H. pylori cells. Therefore, coccoid forms might be important in H. pylori physiology. Consistent with this, Segal and colleagues showed that coccoid forms are able to translocate CagA—one of the major virulence factors and the only known effector protein of the H. pylori type IV secretion system—and induce cellular changes [36]. Coccoid forms express other virulence factors, including the functional CagA. We showed that coccoid forms modulate NF-κB activation. The morphological transition of H. pylori is accompanied by a decrease in the abundance of the GM-tripeptide motif, the hNod1 agonist, and this decrease minimizes the activation of NF-κB (via hNod1) in HEK293T cells and abolishes IL-8 induction in gastric epithelial cells. Thus, the coccoid forms might allow the bacteria to escape or modulate the host response and thereby to persist in the human stomach. To our knowledge, this would be a previously undescribed mechanism for pathogens to respond to a chronic inflammatory response.

Nevertheless, coccoid forms may potentially stimulate epithelial cells via hNod2, in particular in an inflamed mucosa. Indeed, the hNod2 pathway can be induced by TNF-α and INF-γ in an NF-κB-dependent manner [37,38]. During a chronic infection of the gastric mucosa, coccoid forms of H. pylori would preferentially stimulate NF-κB via hNod2. However, hNod2 (as hNod1) senses muropeptides instead of polymeric PG. Muropeptides can be generated either by host lysozyme or by H. pylori endogeneous lytic transglycosylases such as Slt. While lysozyme is abundant in paneth cells, it is almost absent from the mucus layer [39], where H. pylori preferentially resides [40]. Furthermore, like Gram-negative bacteria in general, H. pylori is insensitive to lysozyme's activity. Muropeptides generated by the endogenous lytic transglycosylases such as G(anh)M-dipeptide (Figure 4D) are not sensed by the hNod2 pathway. Hence, coccoid forms are unlikely to be seen by the host, suggesting these could function as a mechanism of escape from and modulation of the host's innate immune system. Campylobacter jejuni also undergoes morphological transition into coccoid forms. C. jejuni usually causes acute gastroenteritis, but a recent study has associated long-term intestinal colonization of patients by C. jejuni with the onset of intestinal mucosa-associated lymphoid tissue lymphoma [41]. Possibly, coccoid forms of C. jejuni are similarly involved in establishing chronic infection.

In conclusion, we report the *amiA* gene as the first genetic determinant to our knowledge discovered that is involved in the transition of spiral bacteria into coccoid forms. Further characterization of AmiA should be of interest in determining how H. pylori regulates the transition from bacillary into coccoid forms and for investigations of the physiological importance, in vitro and in vivo, of this particular bacterial form.

## Materials and Methods

### Bacteria, cells, and growth conditions.

E. coli MC1061 [42] and DH5α were used as hosts for the construction and preparation of plasmids. They were cultivated in Luria Bertani solid or liquid medium supplemented as appropriate with spectinomycin (100 μg/ml) or kanamycin (40 μg/ml) or both. H. pylori strain 26695 [43] was used to construct mutants. PG was extracted from strains 26695 and NCTC11637. H. pylori was grown microaerobically at 37 °C on blood agar plates or in liquid medium consisting of brain–heart infusion (Oxoid, http://www.oxoid.com) with 0.2% β-cyclodextrin (Sigma-Aldrich, http://www.sigmaaldrich.com) supplemented with antibiotic–antifungic mix [44]. H. pylori mutants were selected on 20 μg/ml kanamycin. HEK293T cells were cultured in Dulbecco's modified Eagle's medium containing 10% fetal calf serum. Prior to transfection, HEK293T cells were seeded into 24-well plates at a density of 10^5^ cells/ml as described previously [45].

### Construction of mutants and complementation.

Genes were disrupted as described previously [46]. H. pylori mutants were constructed by allelic exchange after transformation with suicide plasmids or PCR products carrying the gene of interest interrupted by a nonpolar cassette *aphA-3* [46] or mini*Tn*3-km transposon and selected on kanamycin. PCRs were used to confirm that correct allelic exchange occurred. Gene constructions were sequenced to ensure sequence fidelity. All reagents, enzymes, and kits were used according to manufacturers' recommendations. Midiprep (HiSpeed Plasmid Midi Kit) and DNA extraction kits (QIAamp DNA extraction kit) were purchased from Qiagen (http://www.qiagen.com).

The plasmid pILL2000 was used to construct the *amiA* mutant. pILL570 carrying ORF *hp0772* (*amiA* gene) was used as the template for an Expand High Fidelity PCR (Amersham, http://www.amershambiosciences.com) with oligonucleotides 772–1 (5′-gaugaugauggtaccaaggattttaacttcataagtc-3′, in which the underlined sequence corresponds to a *Kpn*I site) and 772–2 (5′-aucaucaucggatccaacacgcagcgattgatcgtctctaac-3′, in which the underlined sequence corresponds to a *Bam*HI site). PCR products were digested with *Bam*HI (Amersham) and *Kpn*I (Amersham) and ligated (T4 DNA ligase, Amersham) with the *aphA-3* nonpolar cassette digested with the same endonucleases.

Complementation experiments were done by insertion of the *amiA* gene in the *rdxA* locus, either in the same orientation or in the reverse orientation. The *amiA* mutant was used as a recipient for the suicide plasmid or PCR products for complementation. Constructs were made as follows. For the same orientation, the construct was made by three-time PCR [47]. Each of three fragments and the final fragment used for transformation were obtained by Expand High Fidelity PCR. First, three fragments were obtained: (i) a 300-bp fragment corresponding to the 5′-end of *rdxA* obtained with oligonucleotides 954F (5′-atgaaatttttggatcaagaaaaaag-3′) and CC772in954–1 (5′-CACAAGCACtacaaattaacctccattgaaatagatgtgcgctgc-3′, with the capital letters corresponding to the sequence hybridizing with the 5′-end of the *amiA* gene); (ii) a 1,320-bp fragment corresponding to the *amiA* gene obtained with oligonucleotides CCrbs772 (5′-gagggttaatttgtagtgcttgtg-3′) and CC772stop (5′-ctaatcattcttgctgaagaaac-3′); and (iii) a 300-bp fragment corresponding to the 3′-end of *rdxA* obtained with oligonucleotides 954Rev (5′-tcacaaccaagtaatcgcatcaac-3′) and CC772in954–2 (5′-GTTTCTTCAGCAAGAATGATTAGtacctggagggaataatgcaatgctatatcgctgtgggg-3′, with the capital letters corresponding to the sequence hybridizing with the 3′-end of the *amiA* gene). The final PCR product was obtained by using a mixture of these three fragments as a template and oligonucleotides 954F and 954Rev.

For the reverse orientation, the pILL570-*rdxA* plasmid was used as the template for an Expand High Fidelity PCR (Amersham) with oligonucleotides 954–2KpnI (5′-cggggtacctacatgcaaaatctctatccg-3′, in which the underlined sequence corresponds to a *Kpn*I site) and 954–1*Bam*HI (5′-cgcggatccgtgtggtaacaactcgctggg-3′, in which the underlined sequence corresponds to a *Bam*HI site). The *amiA* gene was amplified using the following primers: 772-compl-1Bis (5′-cggggatccgagggttaatttgtagtgcttgtgaggttagggg-3′, in which the underlined sequence corresponds to a *Bam*HI site) and 772-compl-2Bis (5′-cgggtaccctaatcattcttgctgaaaaactatcaatgcc-3′, in which the underlined sequence corresponds to a *Kpn*I site). PCR products were digested with *Bam*HI (Amersham) and *Kpn*I (Amersham) and ligated (T4 DNA ligase, Amersham).

The *hp0087* mutant was obtained following natural transformation of H. pylori with a construct made of three PCR products [47]. Each of three fragments and a final fragment used for transformation were obtained by Expand High Fidelity PCR. First, three fragments were obtained: (i) a 300-bp fragment corresponding to the 5′-end of HP0087 obtained with oligonucleotides 87-NotI (5′-ataagaatgcggccgcATGcgttattttcttgtagttttc-3′) and 87-in1 (5′-GTTAGTCACCCGGGTACtgactttcatatctagccatgggg-3′, with the capital letters corresponding to the sequence hybridizing with the 5′-end of the *aphA-3* gene); (ii) a 850-bp fragment corresponding to the *aphA-3* cassette; and (iii) a 300-bp fragment corresponding to the 3′-end of HP0087 obtained with oligonucleotides 87-EcoRI (5′-ggaatTCAattcgcatttaaagggcttg-3′, with the capital letters corresponding to the stop codon of HP0087) and 87-in2 (5′-TACCTGGAGGGAATAATGgactacatccttaaaaacgcc-3′, with the capital letters corresponding to the sequence hybridizing with the 3′-end of the *aphA-3* gene). The final PCR product was obtained by using a mixture of these three fragments as a template and oligonucleotides 87-NotI and 87-EcoRI.

Gamma-glutamyltranspeptidase *(hp1118)* and *hp0771* mutants were obtained by gene interruption with mini*Tn*3; there are no genes downstream from *hp0771* and *hp1118* with the same direction of transcription. The interruption was generated in E. coli DH5α by insertion of mini*Tn*3 into plasmids carrying either *hp0771* or *hp1118* (C. Ecobichon, C. Chevalier, and A. Labigne, unpublished data). Plasmids carrying the insertions were checked by PCR and used to transform H. pylori 26695. Mutants were validated by PCR analysis. The *hp111*8 mutant was also tested for the absence of gamma-glutamyltranspeptidase activity as previously described [48].

### Peptidoglycan extraction and analysis.

Liquid cultures of H. pylori parental strain and isogenic mutant strains were stopped after various times of growth and chilled in an ice-ethanol bath. The crude murein sacculus was immediately extracted in boiling sodium dodecyl sulphate (4% final concentration). Purification steps and high-pressure liquid chromatography (HPLC) analyses were as described previously [49]. Recombinant lytic transglycosylase Slt70 was purified as previously described [50]. Mutanolysin (M1)– or Slt70-digested samples (M1 from Sigma-Aldrich) were analyzed by HPLC on a Hypersil ODS18 reverse-phase column (250 × 4.6 mm, 3-μm particle size) with a methanol (HPLC grade, Fisher Scientific, http://www.fisherscientific.com) gradient from 0% to 15% in sodium phosphate buffer (pH 4.3 to 5.0). Chromatograms were obtained by monitoring at 206 nm. Each peak was collected, desalted, and identified by MALDI-MS as described previously [51].

### Quantification of MurE activity.

Bacteria were collected by centrifugation (3,000 g, 20 min, 4 °C) from 400 ml of culture after 8 h, 24 h, and 48 h of H. pylori growth. The bacterial pellets were washed with potassium phosphate buffer (20 mM, 0.5 mM magnesium dichloride and 2-mercaptoethanol [pH 7.4]), and resuspended in the same buffer. The cells were sonicated with a Branson sonifier (http://www.sonifier.com) at 20W per minute until the lysate was clear. The samples were dialysed twice against the same buffer. MurE activity in these crude extracts was determined as described previously [52].

### Electron microscopy.

Bacteria were washed with PBS (pH 7.4) and stained with ruthenium red or used directly for SEM. For ruthenium red staining, bacteria were prefixed with 2.5% glutaraldehyde, in 0.075% ruthenium red, and 0.1 M cacodylate buffer for 1 h. Samples were rinsed with 0.1 M cacodylate buffer and post-fixed in 1% osmium tetraoxide in 0.1 M cacodylate buffer for 2 h. They were washed in water three times and then dehydrated in a series of ethanol concentrations. Finally, the samples were embedded in Spurr, and ultrathin sections were made. Grids were viewed by TEM with a JEOL Jem 1010 microscope (http://www.jeol.com).

For SEM, samples were washed in PBS, prefixed in 2.5% glutaraldehyde in 0.1 M cacodylate buffer for 30 min, and then rinsed in 0.2 M cacodylate buffer. After post-fixation in 1% osmium tetraoxide (in 0.2 M cacodylate buffer), bacteria were dehydrated in a series of ethanol concentrations. Specimens were critical-point dried using carbon dioxide, then coated with gold and examined with a JEOL JSM-6700F SEM.

### MIC.

To determine the MIC for amoxicillin, suspensions of H. pylori estimated to contain 10^8^ bacteria/ml (OD_600nm_ of 0.1) were serially diluted and grown on plates containing various concentrations of amoxicillin. The MIC was defined as the amoxicillin concentration leading to a decrease of three log of colony-forming units per milliliter as compared to growth without amoxicillin.

### Expression plasmids, transient transfections, and NF-κB activation assays.

The expression plasmid for FLAG-tagged hNod1 was from Gabriel Nuñez (University of Michigan Medical School, Ann Arbor, Michigan, United States) and has been described previously [53]. The expression plasmid for hNod2 was from Gilles Thomas (Fondation Jean Dausset/CEPH, Paris, France). HEK293T cells were used for transfections as described previously [45]. Synergistic activation of NF-κB by PGs, muramyl peptides, and related compounds in cells overexpressing Nod1 or Nod2 was studied as described by Inohara et al. [54]. Briefly, HEK293T cells were transfected overnight with 1 ng of hNod1 or 1 ng of hNod2 plus 75 ng of Ig luciferase reporter plasmid. PG samples (0.1 μg/ml) were digested with 0.25 μg/μl mutanolysin. At the same time, 0.3 μg of PG preparations or 10 pmol of muramyl peptides were added to the cell culture medium, and synergistic NF-κB-dependent luciferase activation was measured after 24 h of co-incubation. NF-κB-dependent luciferase assays were performed in duplicate, and data reported represent at least three independent experiments. Data were standardized with positive controls: *N*-acetylmuramic acid-dipeptide for hNod2 and *N*-acetylmuramic acid-tripeptide for hNod1. hNod1 and hNod2 were activated with H. pylori PG (0.3 μg/ml) digested with M1 or Slt70 as previously described [29].

## Supporting Information

Figure S1 Chromatograms of H. pylori 26695 and NCTC11637 Strains after Two Different Times of Culture (8 h and 48 h) These results are consistent with previous observations [27] and with the results obtained with strain 26695 (Figure 1).
(305 KB PPT)

Figure S2 Schematic Representation of Hypotheses Concerning the Accumulation of GM-Dipeptide in the PG of H. pylori during the Transition from Spiral into Coccoid Forms This accumulation might be generated by the increase in the cytoplasm of PG precursors carrying a dipeptide (then incorporated into periplasmic PG) or by carboxy/endopeptidase activity present in the periplasm (represented by red scissors). The modification of the PG precursor pool in cytoplasm might be due to (i) insufficient *meso*DAP preventing normal biosynthesis or (ii) a decrease of MurE activity, which is then the limiting step in the biosynthesis of precursors and leads to an increase of dipeptide precursor.
(174 KB PPT)

Figure S3 Chromatograms of H. pylori 26695 Isogenic Mutants for *slt, mltD, hp0087,* and *hp1118* after 48 h of Culture The four mutants accumulate the GM-dipeptide at 48 h of growth to the same extent as the parental strain 26695 (see Figure 1).
(273 KB PPT)

Figure S4 MurE Activity in H. pylori Strain 26695 and *amiA* Mutant after 8 h, 24 h, and 48 h of Growth For each time point, the specific MurE activity was measured in crude protein extracts. The specific activity is expressed in nanomoles per minute per milligram.
(40 KB PPT)

Figure S5 Schematic Representation of the *amiA* Locus and *rdxA* Gene The *amiA* gene was inactivated with a nonpolar kanamycin cassette. Complementation studies involved inserting the *amiA* gene into the *rdxA* locus. Note that *amiA* was introduced without a promoter, and, therefore, expression of the *amiA* gene is driven by the *rdxA* locus endogenous promoters. HP0771 was inactivated by a mini*Tn*3-km transposon, since the downstream gene is oriented in the opposite direction.
(44 KB PPT)

Figure S6 H. pylori PG Was Digested with Recombinant Slt70 from E. coli and Analyzed by HPLC Each peak was collected and the structure was determined by MALDI-TOF mass spectrometry. Peaks 1 to 9 correspond to the following anhydromuropeptides: (1) G(anh)M-tripeptide, (2) G(anh)M-tetrapeptide, (3) G(anh)M-tetraglycine-peptide, (4) G(anh)M-dipeptide, (5) G(anh)M-pentapeptide, (6) G(anh)M-tri-tetra-(anh)MG, (7) G(anh)M-tetra-tetraglycine-(anh)MG, (8) G(anh)M-tetra-tetra-(anh)MG, and (9) G(anh)M-penta-tetra-(anh)MG.
(66 KB PPT)

Protocol S1 Supplementary Data (79 KB DOC)

## Abbreviations

(anh)M: *N*-acetyl-anhydromuramic acid
GM-dipeptide: *N*-acetyl-D-glucosminyl-β(1,4)-*N*-acetylmuramyl-L-Ala–D-Glu
GM-tripeptide: *N*-acetyl-D-glucosminyl-β(1,4)-*N*-acetylmuramyl-L-Ala-γ-D-Glu-*meso*-diaminopimelic acid
hNod1: human Nod1
hNod2: human Nod2
HPLC: high-pressure liquid chromatography
km: kanamycin
MIC: minimum inhibitory concentration
*meso*DAP: *meso*-diaminopimelic acid
mtz: metronidazole
PG: peptidoglycan
SEM: scanning electron microscopy
TEM: transmission electron microscopy
