# Supplementary material for: Role of AmiA in the Morphological Transition of Helicobacter pylori and in Immune Escape
Source: PLoS Pathog. 2006 Sep 22;2(9):e97. doi: 10.1371/journal.ppat.0020097 (PMC1574363; doi:10.1371/journal.ppat.0020097)
Supplement: Supplementary file 1 [file ppat.0020097.sd001.doc]

**Supplementary data**

The accumulation of GM-dipeptide in the peptidoglycan of *H. pylori* during the transition from spiral into coccoid forms might be a consequence of an increase in the cytoplasm of the amount of PG precursors carrying a dipeptide (ultimately incorporated in periplasmic PG) or carboxy/endopeptidase activity in the periplasm (represented by red scissors). The modification of the PG precursor pool in the cytoplasm might be due to: i) a failure of *meso*DAP synthesis or ii) a decrease in MurE activity, such that it becomes the limiting step in the biosynthesis of the UDP-MurNAc-tripeptide precursor and leading to an accumulation of the dipeptide precursor. These two alternatives assume that the MraY and MurG proteins can use UDP-MurNAc-dipeptide as a substrate and that the corresponding lipid intermediate can be translocated to the periplasmic side of the cytoplasmic membrane and incorporated into the pre-existing PG.

To elucidate the molecular mechanism of these PG modifications, we studied the effect on GM-dipeptide accumulation in *H. pylori* of adding *meso*DAP (1 mM) to the media. This did not block the accumulation of GM-dipeptide after 48h of growth (data not shown). We concluded that the accumulation of GM-dipeptide was not due to insufficient intracellular *meso*DAP . We then tested whether regulation of MurE activity during the stationnary phase caused the observed PG modifications. Interestingly, MurE activity fell after 48h of culture (Supplementary Fig 3). However, this could not explain the accumulation of GM-dipeptide in the composition of PG *sacculus*, because *E. coli* showed the same decrease of MurE activity (about 50%) **[1]** but the relative amount of GM-dipeptide seemed to decrease and not accumulate in the *E. coli* PG **[2]**. The *amiA* mutant which did not accumulate the GM-dipeptide (see Supplementary Fig 3) showed an equivalent decrease of MurE activity.

**PG hydrolases.** We analyzed PG *sacculus* of various mutants to identify a cytoplasmic or periplasmic carboxy/endopeptidase. Genes of annotated PG hydrolases and/or potential peptidases were used to screen for genes putatively involved in GM-peptide accumulation (blastp in NCBI, http://www.ncbi.nlm.nih.gov/, and **[3,4]**). We also tested whether the putative PG hydrolases were predicted to be periplasmic (SignalP : http://www.cbs.dtu.dk/services/SignalP/ ; and **[4]**).

One of these genes encoded the periplasmic γ-glutamyl transpeptidase (γ-GT, HP1118). This type of proteins can cleave a γ bond after a D-glutamate, as found between the second and the third amino acid in the PG peptide moiety. So, theoretically, the γ-GT could cleave the PG to generate the dipeptide in the PG *sacculus*. Another putative carboxy/endopeptidase was HP0087 which presents limited homology with the NLPC-P60 domain. This domain is present in the p60 protein of *Listeria monocytogenes* which has a PG autolytic activity and was previously predicted to have an endopeptidase activity **[5]**. Moreover, HP0087 was predicted by Tomb and *al.* to be secreted **[4]**, but this was not confirmed by SignalP software (http://www.cbs.dtu.dk/services/SignalP/). Analysis of the PG of these two mutants did not reveal any differences to the parental strain 26695 in terms of muropeptides composition after 8h, 24h and 2 days of culture (supplementary figure 4).

Two genes encode for putative lytic transglycosylases, the *slt* and *mltD* genes. Lytic transglycosylases are periplasmic PG hydrolases and cut in the glycan backbone to generate anhydromuropeptides. Single and double mutants of *slt* and *mltD* were not affected in the accumulation of GM-dipeptide (supplementary figure 4 and Chaput *et al*. manuscript in preparation).

**References**

1. Mengin-Lecreulx D, van Heijenoort J (1985) Effect of growth conditions on peptidoglycan content and cytoplasmic steps of its biosynthesis in *Escherichia coli*. J Bacteriol 163: 208-212.

2. Signoretto C, Lleo MM, Canepari P (2002) Modification of the peptidoglycan of *Escherichia coli* in the viable but nonculturable state. Curr Microbiol 44: 125-131.

3. Boneca IG, de Reuse H, Epinat JC, Pupin M, Labigne A, et al. (2003) A revised annotation and comparative analysis of *Helicobacter pylori* genomes. Nucleic Acids Res 31: 1704-1714.

4. Tomb JF, White O, Kerlavage AR, Clayton RA, Sutton GG, et al. (1997) The complete genome sequence of the gastric pathogen *Helicobacter pylori*. Nature 388: 539-547.

5. Lenz LL, Mohammadi S, Geissler A, Portnoy DA (2003) SecA2-dependent secretion of autolytic enzymes promotes *Listeria monocytogenes* pathogenesis. Proc Natl Acad Sci U S A 100: 12432-12437.
